# Inter-Fractional Variations in Volume and Radiation Dose to the Organs at Risk, High-Risk Clinical Target Volume and Implication of Image-Guided Adaptive Planning During Intracavitary Brachytherapy of Carcinoma Cervix

**DOI:** 10.7759/cureus.21503

**Published:** 2022-01-22

**Authors:** Gurubasappa Patil, Kiran Kumar BR, Geeta Narayanan

**Affiliations:** 1 Radiation Oncology, Vittalrao Tukaram Sutrave Memorial (VTSM) Peripheral Cancer Center, Kalaburgi, IND; 2 Radiation Oncology, Bangalore Medical College and Research Institute, Bangalore, IND; 3 Radiation Oncology, Vydehi Institute of Medical Sciences and Research Centre, Bangalore, IND

**Keywords:** oar, mri, adaptive brachytherapy, inter-fractional variation, intracavitary brachytherapy, carcinoma cervix

## Abstract

Background

Geometrical and anatomical variations occur during the brachytherapy of carcinoma cervix and dose optimization is necessary for every fraction of high‑dose rate intracavitary brachytherapy (HDR-ICBT) for carcinoma of the cervix. A single planned treatment is usually delivered for multiple fractions without consideration of inter-fractional applicator positioning variations and organ motion, which may lead to substantial differences between the planned and delivered doses*. *

Aim and objectives

This study was aimed at evaluating the inter-fractional variation in volume and radiation dose to organs at risk during ICBT for cervical cancer. Furthermore, the doses to high-risk clinical target volume (HRCTV) and the role of adaptive planning in ICBT were assessed.

Materials and methods

Twenty-two patients with carcinoma of the cervix Stage IB2-IVA receiving ICBT were enrolled in the study. All the patients were treated with ICBT four fractions in two applications. For the first application, magnetic resonance imaging-based planning was done, and for the next three fractions, computed tomography (CT) scans were done before every treatment fraction. The CT images were contoured and replanned by keeping the First (I) fraction of each application as the reference. Dose-volume histograms (DVH) were generated, and details of D2cc (DVH on a volume of 2cc) of bladder, rectum, and sigmoid colon (organs at risk-OAR) and D90 HRCTV (dose covering 90%) were documented.

Results

In patients receiving ICBT, variations in OAR D2cc ranged from 1.5 to 2.5Gy for the bladder (p- 0.001), from 2.0 to 3.2Gy (p-0.005) for the rectum and from 1.5 to 3.5Gy for the sigmoid colon (p 0.103). The p-value was significant for D2cc when compared with the OAR volume for the bladder and rectum in both applications, whereas it was not significant for the sigmoid colon. The percentage change in HRCTV coverage was 7% in the first application and by 16% in the second application because of adaptive planning.

Conclusion

Significant variations in doses received by D2cc of the bladder and rectum as well as significant improvement in HRCTV coverage between the fractions were observed because of replanning. Hence, image-guided HDR-ICBT should be incorporated with adaptive planning when delivering in multiple fractions.

## Introduction

Cervical cancer is the second most common cancer among Indian women as per GLOBOCAN 2020 and accounts for one-quarter of the worldwide burden [1]. Cervical cancer is one of the leading causes of mortality, accounting for 17% of all cancer deaths in women aged between 30 and 69 years. It has been reported that more than 85% of the patients are in the age group of ≥40 years and that the maximum number of cases is in the age group of 50-59 years, amounting to 27.37% of all cervical carcinoma cases [2]. External beam radiotherapy (EBRT) with a total dose of 45-50Gy and a dose of 1.8-2Gy per fraction and concurrent chemotherapy with cisplatin followed by brachytherapy (BT) is the standard treatment for locally advanced cervical cancer [3]. BT is a necessary component in the curative treatment of cervical cancers. Low dose rate (LDR) is defined as a dose of 0.4-2 Gray (Gy)/h, and high dose rate (HDR) is defined as a dose of >12 Gy/h. HDR BT has been utilized more frequently than LDR BT over the past two decades as there is no need for the patient to be hospitalized for the treatment, which provides a possible economic advantage. Moreover, the delivery of the treatment in several short fractions may permit greater control over the position of the sources [4].

The American Brachytherapy Society (ABS) has recommended keeping the total duration of the treatment to <8 weeks because further prolongation adversely affects local control and survival. To reduce the overall treatment time and the logistic constraints, multiple fractions are delivered in a single application. A single plan approach for HDR treatments involves contouring and treatment planning at the first fraction and applying the same plan to the remaining fractions. However, this approach does not consider the inter-fractional applicator positioning variations and organ motion that may lead to substantial differences between the planned and delivered doses [5]. A small change in the geometry of the intracavitary brachytherapy (ICBT) applicators may cause a considerable dose difference at various reference points owing to the presence of a large dose gradient in the region close to the BT sources [6,7]. Therefore, calculating the precise dose delivered to the surrounding normal tissues and the clinical target is important for successful treatment.

Significant inter-fraction variations may result from dramatic tumor regression or progression during BT. Thus, replanning with every fraction has been proposed and is currently recommended by the ABS [8-11]. However, a few studies are only available in the literature about the comprehensive method of analyzing the relationship between inter-fractional organ and dose variation that considers both volumetric and organ movement variation. Hence the present study was undertaken.

## Materials and methods

Case selection

This research was a hospital-based prospective single-arm, non-randomized study. Patients diagnosed with squamous cell carcinoma of the cervix for whom definitive chemotherapy-radiation therapy (CTRT) was planned were included in the study. The eligible cases were the patients with histologically proven non-metastatic cervical cancer who were treated with curative-intent definitive CTRT with an EBRT of 46-50Gy in 23-25 fractions with a linear accelerator-based three-dimensional conformal radiotherapy (3DCRT)/intensity-modulated radiation therapy (IMRT) technique along with weekly chemotherapy with cisplatin 40 mg/m^2^. Following completion of the CTRT, ICBT was planned. The HDR BT procedure was performed in accordance with the ABS guidelines.

Contouring and planning

After the procedure, for the first ICBT application, magnetic resonance imaging (MRI) was performed with the applicators and the same used for contouring and planning. GEC ESTRO guidelines [12] were used to contour the high‑risk clinical target volume (HRCTV), bladder, rectum and sigmoid for all patients. Planning was done using Oncentra planning system (Oncentra 4.51 software). The volumes of the HRCTV and organs at risk (OARs) contoured were recorded. Dose-volume histograms (DVHs) were generated, and volume of OARs, D90 and D2cc (DVH on a volume of 2cc) were noted for the bladder, rectum and sigmoid for each treatment. HDR BT was delivered with 192Iridium sources to a prescription dose of 6.5Gy to point A after achieving optimization. The accepted first-fraction treatment plan was used for the 2nd fraction after re-optimization. The 2nd fraction was delivered on the next day. To perform the dosimetric study, the computed tomography (CT) scan was done before delivering the second fraction. Contours were redrawn on the 2nd fraction CT scan by keeping the first-fraction contoured images as a reference. The same method was used for the 3rd and 4th fractions. All the patients were treated with 6.5Gy per fraction with two fractions per application and a total of four fractions in two applications.

Statistical analysis

The statistical analysis was performed using SPSS statistical package version 22 (IBM Corp., Armonk, NY). Descriptive statistics such as mean, minimum and maximum doses, standard deviation for dose received by each OAR was calculated separately for 1st and 2nd application and similar analysis was performed for OAR volumes. To know the significance of data further statistical analysis was performed. Pearson’s correlation was used to find the correlation between whether change in volume of OAR results in change in dose received by OAR and also whether change in volume results in change in D90. Paired t-test was used to determine the significance.

## Results

A total of 22 patients with newly diagnosed squamous cell carcinoma of the cervix were enrolled in the study. Analysis was performed by finding the differences in D2cc of the bladder, rectum and sigmoid for the First(I) application, which included the first and second fractions. The mean, standard deviation, maximum dose, and minimum dose were calculated for the bladder, rectum and sigmoid. A similar descriptive analysis was accomplished for the Second(II) application too.

The maximum and minimum in Table 1 represent the dose received by organs (bladder, rectum, sigmoid) between fractions in single application which can be in +/- from a base line(average) and hence mean lies in the range of maximum and minimum. On average data points are distant from mean by %SD from the mean value hence % of SD calculated using mean and average. Some of the extreme values in data have contributed to larger variation in SD.

**Table 1:** Radiation dose received by HRCTV, bladder, rectum and sigmoid colon. HRCTV: High-risk clinical target volume

Number of Patients Evaluated: 22 Organ	Application	Mean cGy	Standard Deviation	Maximum cGy	Minimum cGy	Percentage of Standard Deviation
Bladder (d 2cc)	I Application	24.89	86.96	163.33	-152.62	+/-16.26
	II Application	-56.10	121.73	208.89	-244.70	+/-23.65
Rectum (d 2cc)	I Application	-18.63	130.26	319.40	-226.52	+/-25.31
	II Application	70.433	137.54	310.25	-198.20	+/-34.50
Sigmoid Colon (d 2cc)	I Application	70.61	90.82	150.99	-186.28	+/-22.78
	II Application	40.32	166.50	342.08	-297.75	+/-47.29
d90 HRCTV	I Application	-31.61	98.59	122.45	-257.32	
	II Application	-82.70	83.01	31.73	-258.64	

The bladder, rectum and sigmoid volumes were noted in the study. The difference in volume for these organs during the 1st application was analyzed. A similar analysis was performed for the 2nd application too. The data were applied for the descriptive analysis of mean, standard deviation, maximum and minimum volumes for the bladder, rectum and sigmoid (Table 2).

**Table 2:** Volume of bladder, rectum and sigmoid colon

Organ	Volume	Mean cc	Standard Deviation (SD)	Maximum cc	Minimum cc
Bladder	I Application	3.10	19.63	43.00	35.70
	II Application	1.36	27.32	51.80	70.20
Rectum	I Application	2.97	11.98	14.40	33.00
	II Application	4.41	18.12	55.10	28.30
Sigmoid	I Application	2.55	20.63	57.66	36.70
	II Application	1.93	16.03	33.10	22.60

The data were employed for determining the correlation of the OAR volumes with D2cc and D90 of high-risk clinical target volume (HRCTV). Pearson correlation test was used for this purpose, and significance was analyzed by applying paired t-test. These tests were used for both applications separately (Table 3). Regression analysis was done to understand the significance of the variables. The variables used were OAR volumes with respect to D2cc and D90 HRCTV (Table 4).

**Table 3:** Paired 2 test (Pearson correlation)

Volume	I Application		II Application	
	D2cc 2 tailed (Pearson correlation)	D90cc 2 tailed (Pearson correlation)	D2cc 2 tailed (Pearson correlation)	D90cc 2 tailed (Pearson correlation)
Bladder	0.012 (0.524)	0.138 (0.327)	0.002 (0.620)	0.789 (0.060)
Rectum	0.244 (0.259)	0.155 (0.491)	0.001 (0.665)	0.764 (0.068)
Sigmoid	0.060 (0.793)	0.327 (0.137)	0.082 (0.082)	0.348 (0.210)

**Table 4:** Regression analysis for significance D90 and D2CC. Sig 2 tailed: This is the two-tailed p-value evaluating the null against an alternative that the mean is not equal to 50.

Regression Analysis	I Application		II Application	
	D2cc p Value	D90cc p Value	D90 sig 2 tailed (Pearson correlation)	D2cc sig 2 tailed (Pearson correlation)
Bladder	.001	.118	.000	.000034
Rectum	.018	.167	.000	.000019
Sigmoid	.163	.087	.006	.000048

## Discussion

Image‑guided BT has replaced the conventional point A‑based dose reporting in gynecological BT in recent years [13-16]. GEC ESTRO GYN working group has published recommendations on target volume definitions and DVH parameters [12,17].

HDR ICBT requires multiple applications, which could lead to inter-fractional variations in the applicator position as well as its spatial position in relation to the pelvic organs, pelvic bony anatomy and the OARs [18-20]. To overcome this drawback, adaptive planning has been implemented in various institutes for HDR BT for cervical cancer. Adaptive planning involves contouring and treatment planning for each fraction, which is facilitated by daily MR images. MRI-guided ICBT allows accurate optimization of the radiation dose and enables the administration of a higher dose to the target tissue. MRI precisely depicts the ICBT probe and any associated complications. The use of MRI for imaging-guided ICBT is expected to increase significantly over the coming years, thereby allowing highly individualized radiation therapy. Imaging modalities with good soft tissue delineation, such as MRI, can be used to assess the tumor response during treatment. By adhering to a uniform bladder filling or emptying routine, it is possible to minimize the variations arising from organ deformation [21,22]. In our study, MR images were used for the first fraction of ICBT planning, while CT images were employed for the second fraction treatment. For dosimetric purposes, the contours were redrawn on the CT images using the first-fraction contours as a reference. The same procedure was followed for the 3rd and 4th fractions too. A multicenter prospective cohort study conducted by Potter et al. on MRI-guided adaptive BT in locally advanced cervical cancer (EMBRACE-I) collected data pertaining to 1341 patients from 24 centers who had undergone MRI-based image-guided adaptive brachytherapy (IGABT). The findings revealed that after a median follow-up of 51 months, the actuarial overall five-year local control was 92%. The actuarial cumulative five-year incidence of grade 3-5 morbidity was 6.8% for genitourinary events, 8.5% for gastrointestinal events, 5.7% for vaginal events and 3.2% for fistulae. The researchers concluded that chemoradiotherapy and MRI-based IGABT result in effective and stable long-term local control across all stages of locally advanced cervical cancer, with limited severe morbidity per organ [23]. Similarly, in our study, MRI-based planning was performed for the first fraction of ICBT.

A study involving 40 patients by Sharma et al. [24] on inter-fractional dose variations in OARs in CT-based BT in locally advanced carcinoma of the cervix evaluated the inter-fractional minimum dose received by the 2cc volumes of the OARs. The first fraction plan was superimposed on the second one to reduce the dosimetric impact. The minimum and maximum D2cc for the bladder, rectum and sigmoid were in the range of 1.3-9.4Gy, 1.4-7.0Gy and 1.6-6.8Gy, respectively, for all fractions. In our study which included 22 patients, for dosimetric analysis, each OAR was recontoured for each fraction. The inter-fractional variations in the dose received by D2cc were 1.5-1.6Gy for the bladder (p 0.001), 2.2-3.2Gy for the rectum (p 0.005) and 1.5-1.9Gy for the sigmoid (p 0.103) during the 1st application. During the 2nd application, variations in the dose received by 2cc bladder, rectum and sigmoid were 2.1-2.5Gy, 2.0-3.1Gy and 3.0-3.5Gy, respectively. Collectively, for all the four fractions, variations in the dose between the fractions for OARs D2cc were in the range of 1.5-2.5Gy for the bladder, 2.0-3.2Gy for the rectum and 1.5-3.5Gy for the sigmoid.

Inter-fractional variations in organ filling and their impact on dosimetry in CT image-based HDR ICBT were examined by Rangarajan [25] which included 170 CT datasets. The volumes of the HRCTV and OAR contoured were recorded for every fraction. DVH were generated, and D90 and D100CTV and D0.1cc, D1cc and D2cc were noted for the bladder, rectum and sigmoid for each fraction. A strong positive correlation was found between the increase in volume and dose (D2cc), which was statistically significant (p = 0.013). In our study, there were a total of 88 data images on change in OAR volume between the fractions. When these data were analyzed in terms of correlation of OAR volume with the dose received by D2cc, a significant uphill positive correlation was noted for the bladder (0.524) and a weak correlation was seen for the rectum (0.259) during the 1st application. On the other hand, during the 2nd application, a positive correlation was seen for the bladder (0.620) as well as for the rectum (0.665) but no correlation was observed for the sigmoid. In case of OAR volume correlation with D90 HRCTV, there was a weak positive linear correlation for the bladder (0.327) and rectum (0.491) for the 1st fraction, while there was no correlation for the second fraction.

Furthermore, statistical significance of OAR volume with dose to D2cc was assessed using regression. The obtained p-value (0.003) was significant for the bladder and rectum in both applications, whereas it was not significant for the sigmoid.

A dosimetric evaluation of using a single treatment plan for multiple fractions in gynecologic BT was conducted by Pinnaduwage et al. [26]. The study comprised one treatment plan for multiple fractions from a single applicator insertion of HDR BT for cervical cancer. Thirteen patients with cervical cancer received the total dose from a single applicator insertion in two fractions that were given at least 6 hours apart within a period of 24 hours. The treatment plan was based on a CT scan taken before the first treatment fraction. A second CT was obtained before the second treatment fraction. The co-registered image series were used to evaluate the dosimetric impact of using a single treatment plan for both fractions. HRCTV coverage was reduced by as much as 17.4 percentage points. This finding is similar to our study involving 22 patients who underwent four fractions of ICBT in two applications, and the treatment plan was based on the first CT scan of each insertion. The percentage change in HRCTV coverage was by 7% for the 1st application and by 16% for the 2nd application. However, when the significance of HRCTV coverage to change in OAR volumes was analyzed using paired t-test, variations in bladder and rectum volume between the fractions showed a significant impact on D90 HRCTV in the 2nd application. However, in the 1st application, there was no significance. This observation could not be explained entirely but could be attributed to the change in applicator position between the fractions.

Considering the inter-fractional variations occurring during ICBT, it is necessary to repeat the imaging process before each fraction to quantify the dose to the target and OAR. Individualized planning with each insertion will be helpful in the accurate estimation of the dose to OAR, failing which inimical effects will be seen in the clinical setting.

As the sample size was less in our study, and these are preliminary results, larger prospective randomized studies with a longer duration of follow-up are needed for strong evaluation of efficacy and to draw inferences about the inter fractional variation in ICBT of carcinoma cervix and the role of image-guided adaptive brachytherapy.

## Conclusions

Our study established the presence of significant variations in doses received by D2cc of the bladder and rectum between the fractions, which may lead to increased toxicity and impact local tumor control. Hence, to lower the toxicity without compromising tumor control, the incorporation of adaptive planning during HDR BT is recommended. Our study also demonstrated the benefit of adaptive planning with respect to improvement in HRCTV coverage. Based on the results, we recommend that image-guided HDR ICBT should be incorporated with adaptive planning whenever ICBT for carcinoma of the cervix is delivered in multiple fractions.

## References

[1] Sung H, Ferlay J, Siegel RL, Laversanne M, Soerjomataram I, Jemal A, Bray F (2021). Global Cancer Statistics 2020: GLOBOCAN estimates of incidence and mortality worldwide for 36 cancers in 185 countries. CA Cancer J Clin.

[2] Rosen VM, Guerra I, McCormack M, Nogueira-Rodrigues A, Sasse A, Munk VC, Shang A (2017). Systematic review and network meta-analysis of bevacizumab plus first-line topotecan-paclitaxel or cisplatin-paclitaxel versus non-bevacizumab-containing therapies in persistent, recurrent, or metastatic cervical cancer. Int J Gynecol Cancer.

[3] Orton CG, Seyedsadr M, Somnay A (1991). Comparison of high and low dose rate remote afterloading for cervix cancer and the importance of fractionation. Int J Radiat Oncol Biol Phys.

[4] Chakraborty S, Patel FD, Patil VM, Oinam AS, Sharma SC (2014). Magnitude and implications of interfraction variations in organ doses during high dose rate brachytherapy of cervix cancer: a CT based planning study. ISRN Oncol.

[5] King GC, Bloomer WD, Kalnicki S (2000). Point dose variations with time during traditional brachytherapy for cervical carcinoma. Med Dosim.

[6] Thomadsen BR, Shahabi S, Stitt JA (1992). High dose rate intracavitary brachytherapy for carcinoma of the cervix: the Madison system: II. Procedural and physical considerations. Int J Radiat Oncol Biol Phys.

[7] Eng TY, Fuller CD, Cavanaugh SX, Blough MM, Sadeghi A, Herman T (2004). Significant rectal and bladder dose reduction via utilization of Foley balloon catheters in high-dose-rate tandem and ovoid intracavitary brachytherapy of the uterine cervix. Int J Radiat Oncol Biol Phys.

[8] Kim RY, Meyer JT, Plott WE, Spencer SA, Meredith RF, Jennelle RL, Salter MM (1995). Major geometric variations between multiple high-dose-rate applications of brachytherapy in cancer of the cervix: frequency and types of variation. Radiology.

[9] Zubizarreta EH, Fidarova E, Healy B, Rosenblatt E (2015). Need for radiotherapy in low and middle income countries - the silent crisis continues. Clin Oncol (R Coll Radiol).

[10] (2015). National Comprehensive Cancer Network: Cervical Cancer. https://www.nccn.org/professionals/physician_gls/pdf/cervical.pdf.

[11] Kim RY, Shen S, Lin HY, Spencer SA, De Los Santos J (2010). Effects of bladder distension on organs at risk in 3D image-based planning of intracavitary brachytherapy for cervical cancer. Int J Radiat Oncol Biol Phys.

[12] Pötter R, Haie-Meder C, Van Limbergen E (2006). Recommendations from gynaecological (GYN) GEC ESTRO working group (II): concepts and terms in 3D image-based treatment planning in cervix cancer brachytherapy-3D dose volume parameters and aspects of 3D image-based anatomy, radiation physics, radiobiology. Radiother Oncol.

[13] Viswanathan AN, Thomadsen B (2012). American Brachytherapy Society consensus guidelines for locally advanced carcinoma of the cervix. Part I: general principles. Brachytherapy.

[14] Dankulchai P, Petsuksiri J, Chansilpa Y, Hoskin PJ (2014). Image-guided high-dose-rate brachytherapy in inoperable endometrial cancer. Br J Radiol.

[15] Okuma K, Yamashita H, Kobayashi R, Nakagawa K (2015). A study of high-dose-rate intracavitary brachytherapy boost for curative treatment of uterine cervical cancer. J Contemp Brachytherapy.

[16] van den Bos W, Beriwal S, Velema L, de Leeuw AA, Nomden CN, Jürgenliemk-Schulz IM (2014). Image guided adaptive brachytherapy for cervical cancer: dose contribution to involved pelvic nodes in two cancer centers. J Contemp Brachytherapy.

[17] Haie-Meder C, Pötter R, Van Limbergen E (2005). Recommendations from Gynaecological (GYN) GEC-ESTRO Working Group (I): concepts and terms in 3D image based 3D treatment planning in cervix cancer brachytherapy with emphasis on MRI assessment of GTV and CTV. Radiother Oncol.

[18] Grigsby PW, Georgiou A, Williamson JF, Perez CA (1993). Anatomic variation of gynecologic brachytherapy prescription points. Int J Radiat Oncol Biol Phys.

[19] Hoskin PJ, Cook M, Bouscale D, Cansdale J (1996). Changes in applicator position with fractionated high dose rate gynaecological brachytherapy. Radiother Oncol.

[20] Kim RY, Meyer JT, Spencer SA, Meredith RF, Jennelle RL, Salter MM (1996). Major geometric variations between intracavitary applications in carcinoma of the cervix: high dose rate vs. low dose rate. Int J Radiat Oncol Biol Phys.

[21] Holloway CL, Racine ML, Cormack RA, O'Farrell DA, Viswanathan AN (2009). Sigmoid dose using 3D imaging in cervical-cancer brachytherapy. Radiother Oncol.

[22] Viswanathan AN, Beriwal S, De Los Santos J (2012). American Brachytherapy Society consensus guidelines for locally advanced carcinoma of the cervix. Part II: high-dose-rate brachytherapy. Brachytherapy.

[23] Pötter R, Tanderup K, Schmid MP (2021). MRI-guided adaptive brachytherapy in locally advanced cervical cancer (EMBRACE-I): a multicentre prospective cohort study. Lancet Oncol.

[24] Mahantshetty U, Krishnatry R, Hande V (2017). Magnetic resonance image guided adaptive brachytherapy in locally advanced cervical cancer: an experience from a tertiary cancer center in a low and middle income countries setting. Int J Radiat Oncol Biol Phys.

[25] Rangarajan R (2018). Interfraction variations in organ filling and their impact on dosimetry in CT image based HDR intracavitary brachytherapy. J Med Phys.

[26] Pinnaduwage DS, Cunha JA, Weinberg V, Krishnamurthy D, Nash M, Hsu IC, Pouliot J (2013). A dosimetric evaluation of using a single treatment plan for multiple treatment fractions within a given applicator insertion in gynecologic brachytherapy. Brachytherapy.

